# The Treatment of Typhoid Fever

**Published:** 1898-11-12

**Authors:** Sidney Phillips

**Affiliations:** Physician to St. Mary's Hospital.


### Hospital Clinics and Medical Progress.

### THE TREATMENT OF TYPHOID FEVER.

Notes from a paper read at the Harveian Society, on November 3rd, by Sidney Phillips, M.D., F.R.C.P., Physician to St. Mary's Hospital.

[Specially Reported for The Hospital, and corrected by the Author.]

Before considering the treatment of typhoid fever note must be taken of the variations in type in the disease. Comparing the earlier literature on the subject with the conditions met with at the present time, and even comparing one's own experience of the disease five-and-twenty years ago with what one sees to-day, one cannot but be struck with the fact that abdominal symptoms are much less pronounced than they used to be. Diarrhœa is less common and severe, while tympanites, ulceration, and the tremor indicated by it, and probably hæmorrhage, are less frequently observed. Indeed, many cases now are constipated throughout the whole course of the illness, and it is not uncommon at the present time to find very slight or no ulceration at a post-mortem examination. Such cases as are fatal must represent many more that have little or no ulceration and recover. In considering the preventive treatment of the disease, proposals which have been made to produce immunity by prophylactic inoculations have not, so far, been extensively adopted in this country, and the means available for the prevention of typhoid fever are practically confined to those sanitary measures by which the proportion of persons dying in London from typhoid has been reduced from 377 per million living in 1869 to 129 per million in 1897. In regard to the results of treatment one has to confess to a certain sense of disappointment. Notwithstanding the advances which have taken place in the treatment of disease in general, and, notwithstanding the improvements in nursing and in the appliances at our disposal, the results of the treatment of typhoid fever, as displayed by statistics, show no improvement over those that were obtained as far back as our statistics will carry us.

Referring to Murchison's tables, we find that, taking a large number of cases, the death-rate was over 18 per cent, and at the present day that is the point at which the death-rate still stands.

The treatment of typhoid fever may best be considered under the heads of the principal dangers which accompany the disease. These arise—(1) From general conditions, such as toxæmia, hyperpyrexia, pyrexia, heart failure, and asthenia; (2) from local lesions special to the fever, such as perforation, hæmorrhage, peritonitis, diarrhœa, constipation, tympanites; and (3) from pulmonary and other intercurrent affections. Toxæmia is one of the main causes of death in typhoid fever, and the main symptoms of the disease are, in fact, the result of a toxæmia, due to the growth of a specific organism, and to the absorption of toxins produced within the intestinal canal; the list of antiseptic substances which have been employed in the hope of lessening the injury so caused is innumerable. For some of these it has been claimed that they can abort the disease, but an abortive treatment of typhoid is not yet known. Many substances have been shown to destroy the bacillus outside the body, and the principle of the antiseptic treatment is no doubt correct. Perchloride of mercury seems to be the most effectual; as a rule it does not cause diarrhœa, its effects on reducing the symptoms and temperature is sometimes very marked. Salol is also useful.

Hyperpyrexia is an extremely uncommon cause of death in typhoid fever, and is but seldom met with except as a complication of some inter-current condition. It is important in its treatment to ascertain that it is not due to some local condition, such as perforation or peritonitis. Otherwise, there is no treatment for hyperpyrexia of much avail except the cold bath. The use of the cold bath as a routine treatment for the ordinary pyrexia of the disease is another matter, even though it may be admitted that cold bathing and cold sponging and other hydro-therapeutic measures have an influence outside and beyond the mere abstraction of heat. Certainly in young and otherwise healthy people, who are so often the sufferers from typhoid fever, the maintenance of a temperature of from 102 deg. to 103 deg. for three weeks or so is not a matter which of itself could do any harm; and when one considers the difficulty, and in many cases the impossibility, of carrying out the "systematic cold bath treatment," and when one bears in mind how large is the proportion of the cases which would recover without it, and how large a number of the remainder would die in any case, one need not be surprised that this treatment has never been adopted as a routine by the mass of the profession; its use would become more frequent if some attempt were made to select cases likely to require it by some other tests than a point in the thermometer. The drugs which may be used to reduce fever are many in number, but it may be said of most of them that they are drugs to be avoided rather than used. Quinine is the best. Antipyrin, phenacetin, and other drugs of that class undoubtedly exercise an injurious influence in any condition of fever tending to malignancy. One common and well-recognised danger in typhoid fever is heart failure. But there is a blood failure as well as a heart failure, and, in fact, what is often spoken of as heart failure is really a condition of asthenia due to lack of blood. The emptiness of arteries after death from typhoid is often notable; hence the great importance of maintaining the nutrition of this important tissue of the body, namely, the bloed.

This brings us to the vital question of the diet. On the one hand, one has to bear in mind the exhausting nature of the disease, and the fact that one of its dangers is a condition of bloodlessness and consequent asthenia; and, on the other, one must not forget how seriously the digestive organs are involved in typhoid fever. The food must be nourishing, but there can be no doubt that, as a rule, while the pyrexia continues the diet should be fluid, and should to a large extent consist of milk. It has been urged that solids can do no harm, seeing that before they arrive at the affected portion of the intestines they are sure to have been reduced to a soft consistency, and that both appetite and digestion are likely to be better if the patient is allowed to follow his own inclination in regard to diet. But it so often happens that disaster of one sort or another follows the taking of solid food when patients, more or less surreptitiously, follow their inclinations in this matter, that it may certainly be taken as a safe rule to keep to liquid food until the temperature has remained at the normal for three days. Longer than this is unnecessary and, indeed, injurious, for the typhoid convalescent has much to make up. It is well to remember, when beginning a more generous diet, to give it at first only on alternate days, so as to be able to hark back if evil should result. Many patients crave for tea. We often enough prescribe caffeine, we prescribe tannin, we give sugar, water, and milk. Why, then, should we withhold these things combined in a cup of tea? As to alcohol, it may, of course, be given if definite reason for its administration should arise, but it should not be given in larger doses than is necessary, and never as a mere routine. It often does harm. The injection of saline fluids into the veins and into the subcutaneous tissues was also spoken of, being occasionally useful in collapse conditions, and the necessity of watchfulness to avoid retention of urine, which might be disastrous, was insisted upon. In the treatment of hæmorrhage, tincture of hamamelis is one of the most efficient means, and the ice-bag, but the latter should not be kept on the abdomen for days together after bleeding has stopped. Although perforation might in extremely rare cases be recovered from, the chances offered by Nature were less than by operation, which was advocated. The difficulty, however, rests in the diagnosis of perforation in many cases.

